# The prominent role of perceptual salience in object discrimination: overt discrimination of graspable side does not activate grasping affordances

**DOI:** 10.1007/s00426-020-01296-2

**Published:** 2020-02-08

**Authors:** Antonello Pellicano, Ferdinand Binkofski

**Affiliations:** Division for Clinical and Cognitive Sciences, Department of Neurology Medical Faculty, RWTH Aachen University, Pauwelsstr. 17, 52074 Aachen, Germany

## Abstract

Responses to object stimuli are often faster when jutting handles are aligned with responding hands, than when they are not: handle-to-hand correspondence effects. According to a location coding account, locations of visually salient jutting parts determine the spatial coding of objects. This asymmetry then facilitates same-sided responses compared to responses on the opposite side. Alternatively, this effect has been attributed to grasping actions of the left or the right hand afforded by the handle orientation and independent of its salience (affordance activation account). Our experiments were designed to disentangle the effects of pure salience from those of affordance activations. We selected pictures of tools with one salient and non-graspable side, and one graspable and non-salient side (non-jutting handle). Two experiments were run. Each experiment had two groups of participants: one group discriminated the location of the salient side of the object stimuli; the other group discriminated the location of the graspable side of them. In Experiment 1, responses were left and right button presses; in Experiment 2, they were left and right button presses plus reach-and-grasp actions. When visual salience was removed from graspable sides, no correspondence effect was observed between their orientation and the responding hands in both the experiments. Conversely, when salience depended on non-graspable portions, a correspondence effect was produced between their orientation and the responding hand. Overt attention to graspable sides did not potentiate any grasping affordance even when participants executed grasping responses in addition to button presses. Results support the location coding account: performance was influenced by the spatial coding of visually salient properties of objects.

## Introduction

As we interact with our environment, our efficiency in coordinating our behavior depends on the recognition of the most salient cues. In part, some of our actions like reaching for objects are based on attentional cues provided by intrinsic, as well as relational properties of objects. There is consolidated evidence that shifts of attention to the location of visual objects automatically generates response codes (Simon 1990). However, motor response codes are also generated by objects themselves when attention is shifted to one of their components (Anderson et al. 2002). Experimental evidence of such motor activations have been provided through the so-called handle-to-hand correspondence effect, which states that when common-use objects (e.g., kitchen or garage tools) are presented in the observer’s visual field and with their handle protruding left- or rightwards, hand responses (e.g., button presses) are faster and more accurate when the handle orientation corresponds to the location of the response, compared to when there is no spatial correspondence between them. Anderson et al. (2002) first argued that the visual asymmetry of an object, as produced by its jutting handle, is likely to induce an attentional bias to it, which in turn generates a spatially corresponding motor response. The representation of such motor response is not limb specific, that is, it is unrelated to the effector that produces left/right responses but can emerge, for example, also with foot responses (Phillips and Ward 2002). This explanation is well served by theories of attention that suppose a link between motor programming and attentional control (Stoffer and Umiltà 1997).

In the wake of the original idea of Anderson et al. (2002), more recent works have provided evidence in favor of a location coding account for spatial correspondence effects obtained with visual objects (Cho and Proctor 2010). Accordingly, the graspable parts of depicted objects, as they most frequently protrude on one side, become perceptually salient to the observer. Indeed, salience is a direct product of perceived asymmetry in the stimulus image, which renders one side of the depicted object more spatially distinctive than the other (Cho and Proctor 2011). The location of these salient portions are coded within a spatial stimulus set (e.g., left and right stimulus spatial codes) that overlaps (Kornblum et al. 1990) with the spatial response set (left and right responses), thus setting the preconditions for the emergence of a stimulus–response (S–R) spatial correspondence effect. In other words, the handle-to-hand correspondence effect would correspond to an object-based Simon effect (Cho and Proctor 2010), that is, to the earlier evidence that stimulus location (or orientation) can influence motor responses (e.g., button presses) even when task irrelevant (Scorolli et al. 2015; Pellicano et al. 2010b; Vu et al. 2005; see Proctor and Vu 2006 for a review).

However, the handle-to-hand correspondence effect has been originally explained in the context of the affordance activation hypothesis (Tucker and Ellis 1998) and independently of the location coding of salient stimulus components. Accordingly, the perception of an object tool, beyond the extraction of its perceptual features such as color, shape, size, and location orientation, also activates appropriate grasping actions which are consistent with its identity and its canonical use (e.g., Bub and Masson 2010; Goslin et al. 2012; Pellicano et al. 2010a). Indeed, different from the previously described account, activated actions are limb specific: they will involve the left or the right hand depending on the left- or rightward orientation of the object handle (variable affordances; see Pellicano et al. 2011; Pellicano et al. 2017a). As a result, responses to a graspable object become faster and more accurate when the responding hand is aligned with the left–right orientation of its graspable part (i.e., the handle), compared to when it is not.

Evidence has been provided against this affordance activation account and in favor of a location coding account (see Proctor and Miles 2014 for a review). However, some other studies suggested that affordance activation effects can be dissociated from abstract location coding effects. For example, Buccino et al. (2009) performed a transcranial magnetic stimulation (TMS) study employing pictures of objects with intact and broken handles, and with graphic symbols, all providing similar lateral asymmetry. When symbols and objects handles were rightward oriented, motor evoked potentials (MEPs) collected from the right hand were larger with intact handles than with broken handles and symbols. Cardellicchio Sinigaglia and Costantini (2011) also performed a TMS study recording (MEPs), while participants observed graspable and non-graspable objects located within or outside their own reachable space. They found larger MEPs with reachable graspable objects compared to non-graspable objects, and non-reachable graspable objects. These two studies claimed that the recruitment of the motor system is spatially constrained and depends on the integrity of perceived objects. Pappas (2014) observed that more realistic representations of graspable objects (photographs instead of silhouette objects) and between-hands button press responses (i.e., effector specific) can activate grasping affordances. A follow-up study by Proctor, Lien, and Thomson (2017) provided instead evidence of a location coding of salient features, but also evidence suggestive of a late contribution of an effector-specific correspondence component with photograph images that may be consistent with affordance activations.

In a recent study, Pellicano et al. (2017b) used two sets of object stimuli with specific structural characteristics that allowed to separate the effects of the graspable portion from those of the visually salient one and vice versa, and to unambiguously attribute the resulting correspondence effect to location coding or to the activation of motor affordances. For their creamer stimulus set, the only goal-directed portion (the spout) was left–rightward jutting (and visually salient). No jutting handle was present and their graspable part was the portion of the whole body that was opposite to the goal-directed one. For their teapot stimulus set, the spout was also the left–rightward jutting portion, but a jutting handle was provided on the central–upper part of the object. A set of seven experiments was performed that clearly showed a spatial correspondence effect between the lateralized button press responses and the location of the spout. Thus, when salience was not a property of the graspable parts of the objects anymore, and the same objects appeared asymmetric because of another spatially distinctive portion, correspondence effects were driven by this second portion, thus clearly supporting the location coding account for object-based spatial correspondence effects (see also Pellicano et al. 2018a). This is also the case when an object stimulus is centered on the screen so that its base or most of its body protrudes on the left/right rather than its handle (Pellicano et al. 2018b; Proctor et al. 2017). Moreover, Pellicano et al. (2017b) proposed an action coding account that refined the original location coding account claiming that the spatial coding of object tools depends on a higher-level process that implies evaluation of semantic and action features, instead of lower-level processing of structural asymmetries in objects body. Indeed, the spatial direction of the action proper of a perceived object is coded (e.g., a rightward pouring action for creamers and teapots with their spout on the right; see Pellicano et al. 2017b, Experiment 4). This account also finds support in the notion of tool-centered mechanical actions proposed by Osiurak et al. (2017) to address the relation between a tool that performs an action and an object that “receives” it. Mechanical actions are distinct from hand-centered affordances that are instead action possibilities between a body/hand and a tool. Furthermore, Osiurak et al. (2017) defined a third domain of tool-centered contextual relationship that is also independent of affordances and identifies the semantic relationships between two or more tools (e.g., a creamer and a plate). Thus according to this view, the objects pairs utilized in Pellicano et al. (2017b) would also fall within this third domain (but see also Borghi 2018; Zipoli Caiani and Ferretti 2017 for an overview of semantic and pragmatic interactions in vision for actions).

In Pellicano et al. (2017b) experiments, the location of the graspable and of the goal-directed portions of stimuli was always irrelevant to the task. Instead, a recent study by Xiong et al. (2019) tested the affordance activation hypothesis when the horizontal orientation of tools (images of spoons and bamboo chopsticks) was made relevant in a button press choice reaction task. In their experiments, the handle side was made more salient than the tip side (i.e., spoons head and pointed side of chopsticks), and vice versa. Participants were instructed to respond to the location of the handles or of the tips by using a compatible stimulus–response mapping (i.e., press the button on the same side as the handle/tip) or an incompatible mapping (i.e., press the button on the opposite side as the handle/tip). Results displayed that the salient part of the tools, either the handle or the tip, could not be entirely ignored to attend solely the less salient part, a result that well fitted with the location coding account of object-based correspondence effects.

In the present study, we applied Xiong et al. (2019)’s approach to follow up Pellicano et al. (2017b) investigation on spatial coding/affordance activation. The graspable and the goal-directed portions of their objects stimuli were turned relevant for the task. Compared to Xiong et al. (2019), we employed a set of six creamer stimuli (Pellicano et al. 2017b, Experiment 1A), instead of one stimulus only per experiment, whose structure maximized the difference in salience between the graspable and the goal-directed sides to the advantage of the latter, while keeping the graspable side correctly identifiable (see introduction to Experiment 1). Thus, the structure of these stimuli would basically exclude any relation of a handle-to-hand correspondence effect with a visual salience bias. According to a recent definition, affordances are relations between the features of a situation and the abilities of an individual; therefore to perceive an affordance, one individual must have the ability to perceive that one situation (including objects within the environment) supports and perhaps demands a certain kind of action (Chemero 2009). Indeed, to request participants to overtly process the graspable part of one object would “situate” the object so that its perception would more strongly demand a proper action; in other terms, it would plausibly increase the chances for a grasping action to be activated. In Experiment 1, button press responses of the two hands were required as for the original experiments in Pellicano et al. (2017b). In Experiment 2, a reach-and-grasp action was performed immediately after the button presses to further facilitate the observation of affordances activation.

## Experiment 1

Different from our previous investigations (Pellicano et al. 2010a, b; 2017b; 2018a; 2018b), for the employed object stimuli the location of their graspable or visually salient portions were task relevant. The same creamers stimulus set as Pellicano et al. (2017b)—Experiment 1A was employed. Visual salience was systematically removed from the graspable side of these objects, whereas their own functional meaning was preserved (Bub et al. 2008). This latter point was originally assessed in Pellicano et al. (2017b) on a separate group of participants who were presented with the pictures of the creamers and asked to reproduce an appropriate grasping gesture. They scored a high and positive correlation between the expected lateral power grasp and the grasp they performed spontaneously (e.g., the right hand ideally around the rightward oriented graspable portion). Furthermore, in the present study, a real creamer with the same characteristics as the picture stimuli was presented to the participants before the start of the experiment. When required to show the experimenter its proper use, participants all grasped the creamer to perform the correct functional action associated with it (i.e., they grasped the creamer from the correct side and mimicked to pour milk through the spout). Both these evidences clearly suggested that a “handle” was correctly identifiable for our creamer stimuli and, plausibly even if not typically jutting, it would not reduce the chances to automatically activate a grasping affordance.

One group of participants was instructed to attend to the location of the spout to discriminate the horizontal orientation of the creamers, whereas the other group was instructed to attend to the orientation of their graspable side. Participants completed a choice-reaction task in which they responded to the location of one (goal-directed or graspable) side of the object with a spatially corresponding response or a spatially non-corresponding response. On the one hand, according to the location coding account, faster and more accurate performance was expected in the goal-directed instruction group when the location of the spout corresponded to the location of the response than when it did not (Pellicano et al. 2017b). However, the same effect was expected in the graspable instruction group: notwithstanding the instructions to focus on the graspable side and rely the performance on its location, it would be the most salient side that still drives the spatial coding of the objects and biases performance, accordingly. On the other hand, if instructions to rely on the graspable portion instantiated grasping affordances, as opposite to what observed when the graspable portion was task irrelevant (Pellicano et al. 2017a, b), faster performance would be observed when the responding hand was aligned to the graspable side of the creamers, than when it was not. Using G*Power (Version 3.1.9.2; Faul et al. 2007), we estimated that, given an *α* level of 0.05, with a sample of 12 participants for each instruction group, we would have 95% power to detect an effect size of 0.68 (based on Pellicano et al. 2017b—Experiment 1A).

### Method

#### Participants

Thirty-six students from RWTH Aachen University received 5 euros for their participation. All had normal or corrected-to-normal vision, normal color vision, were right-handed according to the Edinburgh Inventory of Handedness (Oldfield 1971), and naïve as to the purpose of the experiment. Eighteen students were instructed to discriminate the goal-directed side of the stimuli (9 females, 9 males; mean age 26.2 years; SD 4.4 years; + 86.8/100 handedness score), whereas the other 18 were instructed to discriminate their graspable side (12 females, 6 males; mean age 25.1 years; SD 5.7 years; + 83.1/100). Participants were randomly assigned to the two groups. The present experiment and the following one were conducted according to the Convention of Helsinki. The Ethics Committee of the Medical Faculty at RWTH Aachen University approved the study and all participants gave their informed consent before participating.

#### Materials

Participants seated facing a monitor with a 17″ screen (1024 × 768 resolution) driven by a 3-GHz PC. The monitor was placed on a small platform 16 cm higher than the table top with the horizontal midline of the screen being at 41 cm from it. Stimulus presentation, response timing, and data collection were controlled by the E-Prime Professional v2.0 software (https://www.pstnet.com). A PST serial response box was connected to the PC and employed as the response device.

**Fig. 1:**
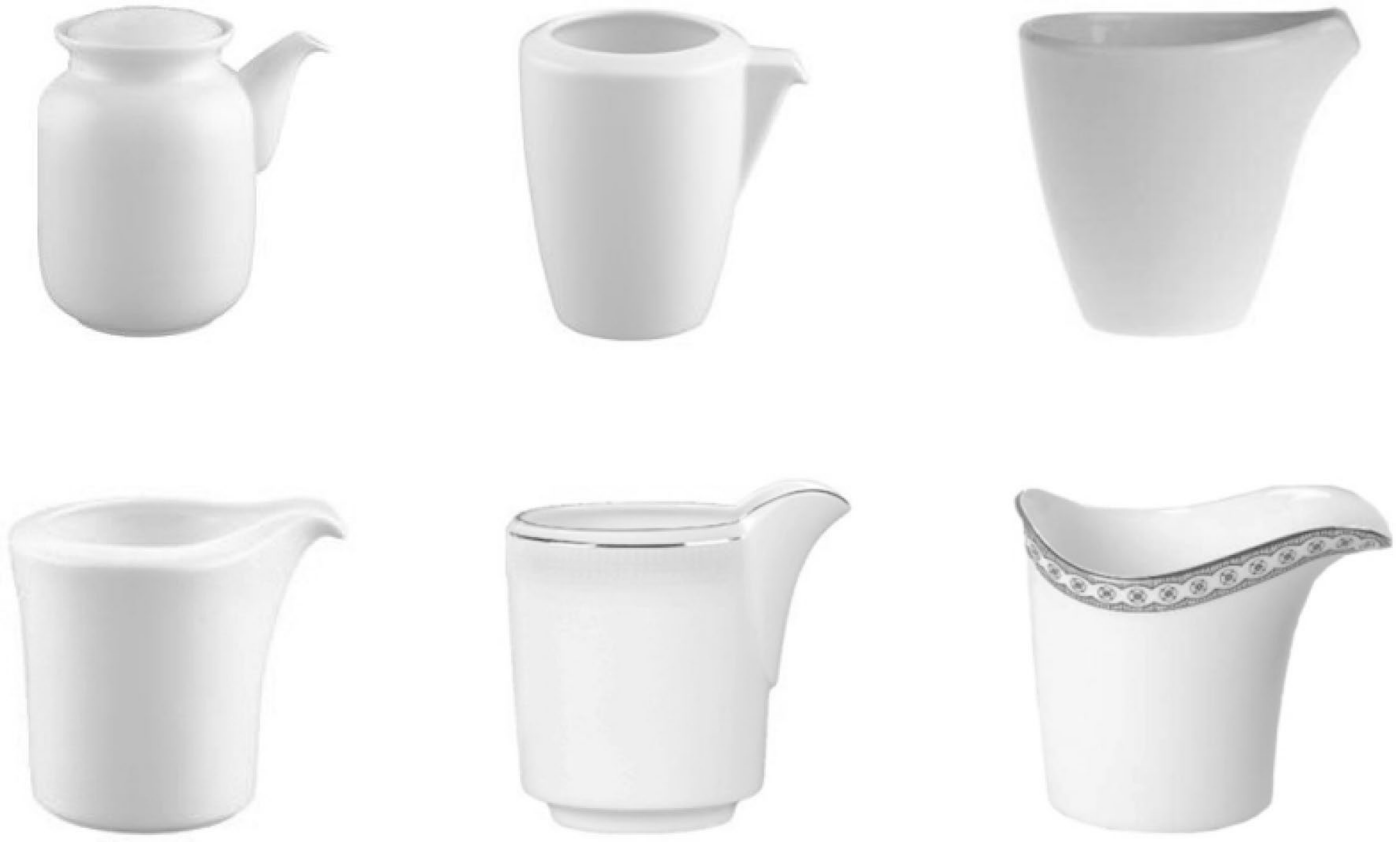
The creamers stimulus set was the same as the one used in Pellicano et al. (2017b)

Participants viewed the stimuli from a distance of approximately 60 cm. A black fixation cross (0.4 × 0.4 degrees of visual angle) and the target stimulus were presented at the center of the screen and on a white background. The response box was centered on the vertical midline of the screen in front of the participant. Six pictures of creamers all sharing a jutting spout on one side and a non-jutting graspable portion on the opposite side were employed as the visual stimuli (Fig. 1). Stimuli ranged from 6° to 7.6° in width, and from 7.2° to 7.6° in height; they were presented in upright orientation, and centered on the base of the main body of the creamer, so that the only left–rightward jutting part was their spout. Each creamer was displayed with the spout on the left side and the graspable portion on the right side in half the trials, and with the spout on the right side and graspable portion on the left side in the other half.

#### Procedure

The experiment took place in a dimly lit and noiseless room. At the beginning of each trial, the fixation cross was presented for 1000 ms, followed by the target stimulus, which remained on screen for a maximum of 1000 ms or until a response was produced. Responses were left and right button presses (“1” or “5” of the response box) with the left or right index finger, respectively. For one group of participants, the goal-directed portion (i.e., the spout) was the task-relevant feature of the stimuli. Two blocks of trials were arranged according to different S–R assignments (i.e., mappings). In the compatible mapping block, participants were instructed to press the button which resulted on the same side as the goal-directed portion of the creamers, whereas in the incompatible mapping block participants had to press the button opposite to the goal-directed portion of the creamers. For the other group, the graspable portion was the task-relevant feature of the stimuli. Therefore, in the compatible mapping block, participants pressed the button on the same side of the graspable portion of the creamers, whereas in the incompatible mapping block they had reversed S–R assignments. The order of compatible and incompatible blocks was counterbalanced across participants for both the groups.

**Fig. 2:**
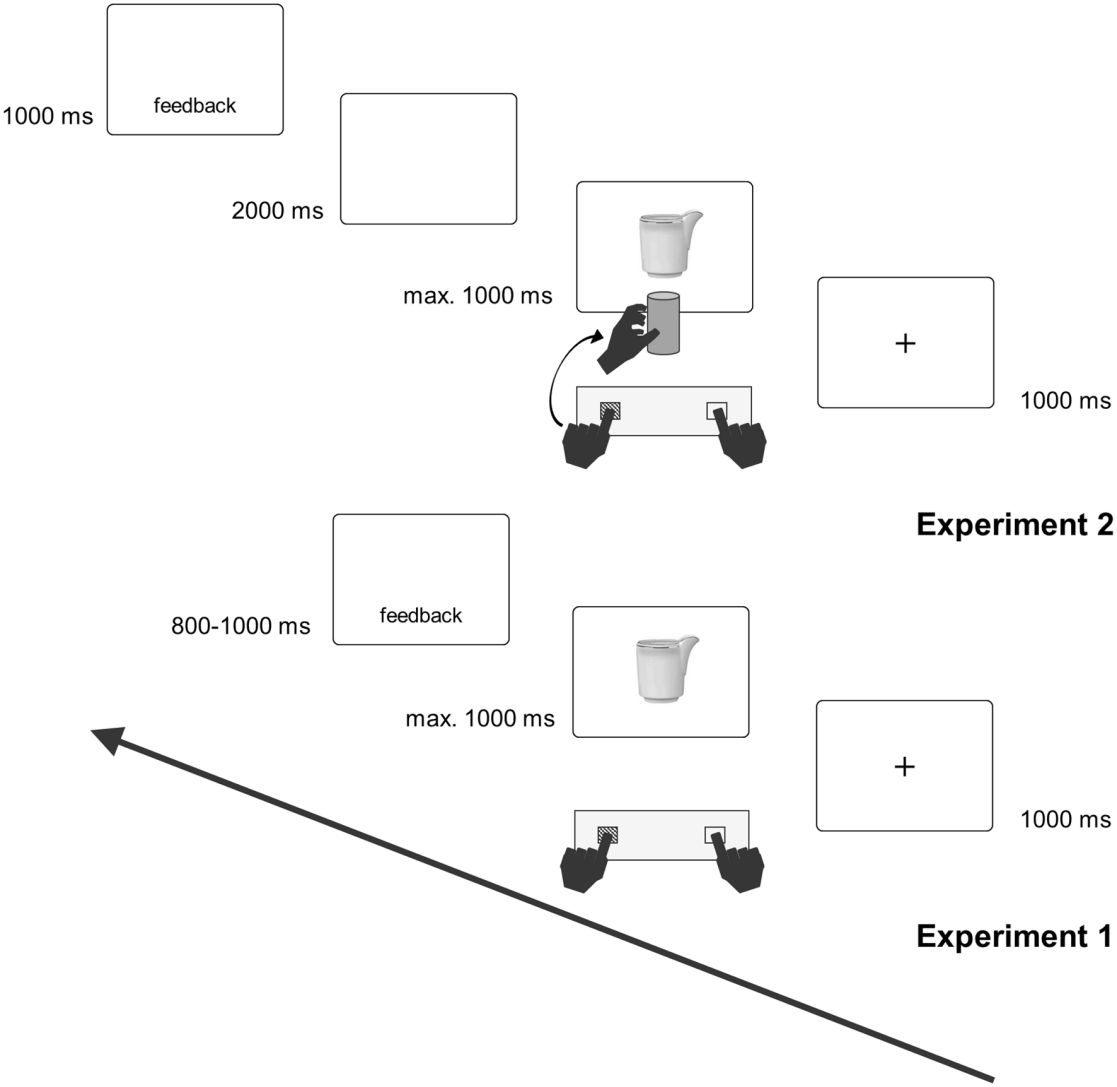
Trial structure in Experiment 1 and Experiment 2. The sequences represent a compatible response for the grasping instruction groups, and an incompatible response for the goal-directed instruction groups

At correct responses, the actual RT was displayed in the middle–bottom part of the screen for 800 ms. If an incorrect or no response occurred, the word “FALSCH” (wrong) or “FEHLT” (missing) was provided for 1000 ms together with a low pitch tone. Each stimulus was displayed four times within one block of randomized trials. Each block had 240 trials with a short break in between, and was preceded by 12 training trials. In total, 480 experimental trials were administered for each participant; the total duration was about 30 min. At the end of the experiment, participants were asked to report the response selection strategies they applied in both their compatible and incompatible mapping blocks (Fig. 2).

#### Design

For the goal-directed instruction group, compatible responses were mapped to the same side as the spout of the creamers, whereas incompatible responses were mapped to the opposite side as the spout. For the graspable instruction group, compatible and incompatible responses were mapped to the same side, and to the opposite side as the graspable portion of the creamers, respectively.

Mean correct RTs and arcsine-transformed error rates (ERs) were submitted to two separate analyses of variance (ANOVAs) with instructions (goal-directed vs. graspable) as between-subject variable and compatibility (compatible vs. incompatible mappings) as within-subject variables. All statistical tests were performed in SPSS (IBM, USA). When necessary, paired and independent samples *t* tests were performed as post hoc comparisons with Bonferroni corrected *p* value. An open-source tool was used to compute Cohen’s *d*_z_ effect size for the *t* tests (https://memory.psych.mun.ca/models/stats/effect_size.shtml).

## Results

For each participant, omitted responses (0.05%), RTs shorter than the overall individual mean − 2 standard deviations (0.4%) or + 2 SD (3.7%) were excluded from the analyses.

### RTs

**Fig. 3:**
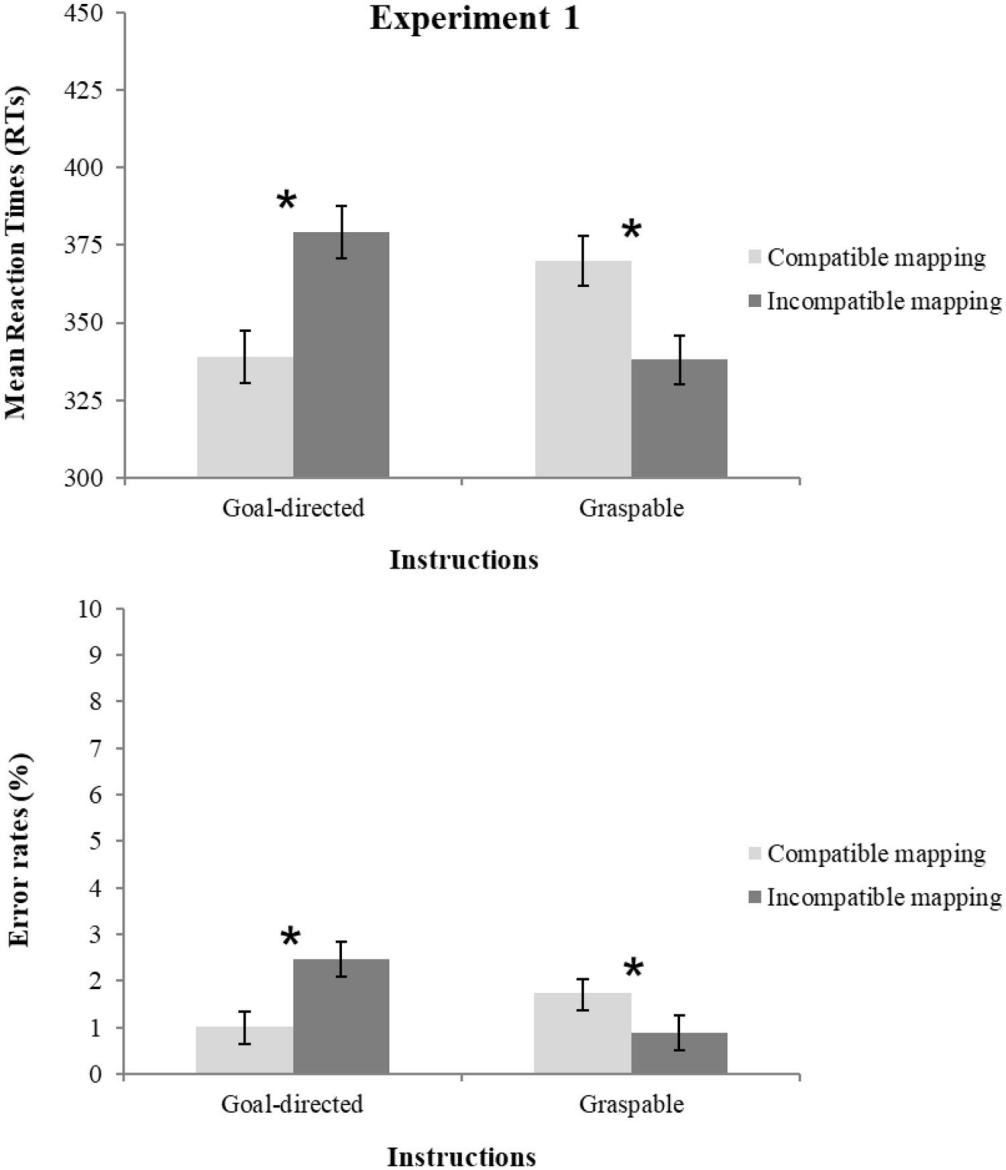
Mean reaction times (RTs, upper panel) and error percentages (ERs, bottom panel) of Experiment 1 as a function of compatibility and instructions (goal directed vs. graspable)

The main effect of instructions was not significant, *F*(1, 34) = 0.218, *p* = 0.644, *η*^2^_p_ = 0.01, as well as the main effect of compatibility, *F*(1, 34) = 1.718, *p* = 0.199, *η*^2^_p_ = 0.05. The interaction of instructions and compatibility was significant, *F*(1, 34) = 129.693, *p* < 0.001, *η*^2^_p_ = 0.79 (see Fig. 3). The goal-directed instruction group displayed shorter RTs for salient portion-to-hand compatible (339 ms) than for incompatible mapping trials (379 ms), *t*(17) = 10.981, *p* < 0.001, *d*_z_ = 2.59 (all participants showed their effect in the same direction as the group effect). The graspable instruction group instead displayed a reversed effect: longer RTs for graspable portion-to-hand compatible (370 ms) than for incompatible mapping (338 ms), *t*(17) = 6.176, *p* < 0.001, *d*_z_ = 1.45 (17 out of 18 participants showed their effect in the same direction as the group effect) (Bonferroni-corrected alpha level = 0.025). Thus, the compatibility effect was significantly driven by the location of the spout and not by the opposite task-relevant graspable side. The absolute sizes of the compatibility effects in the two groups did not differ significantly, *t*(34) = 1.311, *p* = 0.199, *d*_z_ = 0.43 (see Table 1).

**Table 1:** Mean reaction times (RTs) and mean percentage of errors (ERs) with standard deviation in brackets, for compatible and incompatible mapping trials in the goal-directed and graspable instruction groups

Experiment 1	Instructions			
	Goal directed		Graspable	
Mapping	RTs (s.d.)	ERs (s.d.)	RTs (s.d.)	ERs (s.d.)
Compatible	339 (29.7)	1.02 (1.2)	370 (40.3)	1.7 (1.4)
Incompatible	379 (36.1)	2.5 (1.9)	338 (31.1)	0.9 (1.2)
Compatibility effect	40*	1.5*	− 32*	− 0.9*

Compatibility effect was computed as the difference in RTs and ERs between incompatible and compatible mapping trials.

*Significant differences

### ERs

Errors averaged 1.5% of total trials. The main effects of instructions and compatibility were not significant, *F*(1, 34) = 0.953, *p* = 0.644, *η*^2^_p_ = 0.03, and *F*(1, 34) = 2.809, *p* = 0.103, *η*^2^_p_ = 0.08, whereas their interaction was significant, *F*(1, 34) = 41.513, *p* < 0.001, *η*^2^_p_ = 0.55 (see Fig. 3). Consistent with the RT data, the goal-directed instruction group was less error prone in the salient portion-to-hand compatible (1%) than in the incompatible mapping condition (2.5%), *t*(17) = 5.092, *p* < 0.001, *d*_z_ = 0.91, whereas the graspable instruction group was more error prone in the graspable portion-to-hand compatible (1.7%) than in the incompatible mapping condition (0.9%), *t*(17) = 3.948, *p* = 0.0010, *d*_z_ = 0.67 (Bonferroni-corrected alpha level = 0.025). Thus similar to RT data, a reversed compatibility effect resulted when graspable instructions were given. The absolute sizes of the two compatibility effects did not differ significantly, *t*(34) = 1.675, *p* = 0.103, *d*_z_ = 0.54 (see Table 1).

## Experiment 2

In Experiment 2, the response set was modified: after the button press with the left or right hand, and participants had to perform a reach-and-grasp movement with the same hand. Indeed, their performance implied the programming and the execution of actions that would better foster the activation of affordances compared to simple button presses, as they were more similar to (or even reproduced exactly) the kind of manipulation typically associated with the perceived objects. Recently, Roest et al. (2016) had participants that performed a bimanual choice-reaction task to discriminate the color or the vertical orientation of a beer mug with the handle oriented on the left or on the right. A handle-to-hand correspondence effect was observed when participants responded by grasping a cylinder (see also Bub and Masson 2010), but also when they responded by touching a screw. Furthermore, the effect disappeared when a unimanual go/no-go task was given. They concluded that overlap between the grasping action associated with the objects handle and the grasping action executed as response did not play a role in the observed correspondence effect. Rather, the effect was due to competition at response selection stage based on task demands or abstract spatial codes. Similarly in Pellicano et al. (2018a), participants performed either a unimanual go/no-go task (absence of response alternatives) or a joint go/no-go task (available response alternatives) both with grasping and button press responses to object pictures with lateral handles. They found no handle-to-hand correspondence effect in the individual go/no-go task either when a grasping or a button press response was required, whereas a significant effect emerged in the joint go/no-go task, irrespective of response modality. These results were inconsistent with activation of limb-specific and response alternative-independent affordances, and rather supported the location coding account.

Thus, these studies displayed that the use of grasping responses failed to favor the activation of grasping affordances when the task-relevant features of stimuli were action unrelated like color or vertical orientation. In Experiment 2, we investigated whether to overtly process the graspable part of objects, together with performing grasp responses, was more likely to activate knowledge about the motor actions typically applied to the perceived objects. If so, different from Experiment 1, a positive spatial compatibility effect should be observed in the group of participants instructed to respond to the location of the graspable portion of stimuli.

### Method

#### Participants

Forty students from RWTH Aachen University received 5 euros for their participation. None of them also participated in Experiment 1. All had normal or corrected-to-normal vision, normal color vision, were right-handed (Oldfield 1971), and naïve as to the purpose of the experiment. Twenty students were instructed to discriminate the goal-directed side of the stimuli (13 females, 7 males; mean age 25.9 years; SD 6.9 years; + 81.3/100 handedness score), whereas the other 20 were instructed to discriminate their graspable side (10 females, 10 males; mean age 25.3 years; SD 4.1 years; + 86.7/100). Participants were randomly assigned to the two groups.

#### Materials, procedure and design

Materials, procedure and design were the same as in Experiment 1 except for what follows. A metal cylinder (100 mm in height, 47 mm of diameter) was vertically placed at 4 cm distance from the screen surface, and centered on the vertical midline of it. Responses were left and right button presses (“1” or “5” of the response box) with the left or right index finger, followed by a reach-and-grasp movement toward the cylinder (Fig. 2). For the goal-directed instruction group, in the compatible mapping block, participants were instructed to press the button which resulted on the same side as the creamers’ goal-directed side, and then to reach and grasp the cylinder with a lateral power grasp of the same hand (e.g., spout on the right—right button press with the right index finger and power grasp of the cylinder with the right hand from the right side), whereas in the incompatible mapping block participants had to press the button opposite to the goal-directed side of the creamers and reach and grasp the cylinder also from the opposite side (e.g., spout on the right—left button press with the left index finger and power grasp of the cylinder with the left hand from the left side). For the graspable instruction group, in the compatible mapping block, participants pressed the button on the same side of the graspable portion of the creamers and then reached and grasped the cylinder with the same hand and a lateral grasp (e.g., graspable portion on the right—right button press with the right index finger and power grasp of the cylinder with the right hand from the right side), whereas in the incompatible mapping block they had reversed stimulus–response assignments (e.g., graspable portion on the right—left button press with the left index finger and power grasp of the cylinder with the left hand from the left side). We assumed that the reaction times measured at the button press should be affected by the programming of a reach-and-grasp response to be executed after the button press (Roest et al. (2016, see also Rubichi and Pellicano 2004), thus allowing to measure eventual effects of affordance activations.

To avoid overlaps with the reach-and-grasp action, no feedback was given immediately after correct button presses. A 2000 ms blank screen was displayed after the button press response to allow the execution of the reach-and-grasp action before the feedback display: if an incorrect or no button press response occurred, the word “FALSCH” (wrong) or “FEHLT” (missing) was provided for 1000 ms together with a low pitch tone (see Fig. 2).

**Fig. 4:**
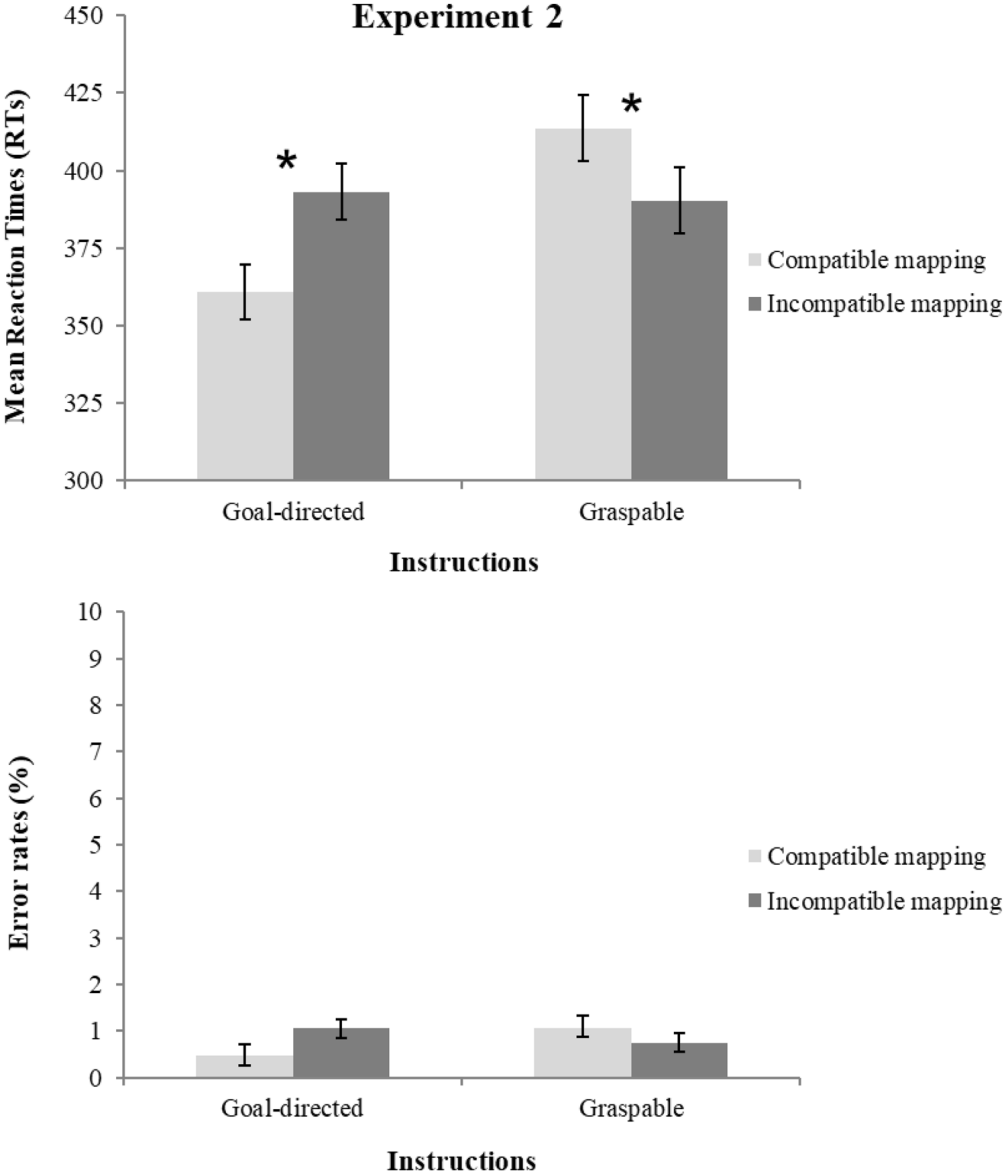
Mean reaction times (RTs, upper panel) and error percentages (ERs, bottom panel) of Experiment 2 as a function of compatibility and instructions (goal directed vs. graspable)

For RTs, the same analysis of Experiment 1 was performed with the same statistical model. Error rates averaged less than 1% of the total trials (0.8%) and were not analyzed; for the sake of completeness, they are nevertheless reported in Fig. 4 and Table 2.

**Table 2:** Mean reaction times (RTs) and mean percentage of errors (ERs) with standard deviation in brackets, for compatible and incompatible mapping trials in the goal-directed and graspable instruction groups

Experiment 2	Instructions			
	Goal-directed		Graspable	
Mapping	RTs (s.d.)	ERs (s.d.)	RTs (s.d.)	ERs (s.d.)
Compatible	361 (33.1)	0.5 (0.6)	414 (45.97)	1.1 (1.5)
Incompatible	393 (34.5)	1.1 (0.9)	390 (58.3)	0.8 (0.9)
Compatibility effect	32*	0.6	− 24*	− 0.3

Compatibility effect was computed as the difference in RTs and ERs between incompatible and compatible mapping trials.

*Significant differences. As mean ERs were overall less than 1%, they were reported in the table but not analyzed

## Results

For each participant, omitted responses (0.2%), RTs shorter than the overall individual mean − 2 standard deviations (0.3%) or + 2 SD (4.02%) were excluded from the analyses.

The main effect of instructions was not significant, *F*(1, 38) = 3.657, *p* = 0.063, *η*^2^_p_ = 0.09, as well as the main effect of compatibility, *F*(1, 38) = 0.822, *p* = 0.370, *η*^2^_p_ = 0.02. The interaction of instructions and compatibility was significant, *F*(1, 38) = 31.672, *p* < 0.001, *η*^2^_p_ = 0.45 (see Fig. 4). The goal-directed instruction group displayed shorter RTs for salient portion-to-hand compatible (361 ms) than for incompatible mapping trials (393 ms), *t*(19) = 5.901, *p* < 0.001, *d*_z_ = 1.32 (all participants showed their effect in the same direction as the group effect). The graspable instruction group instead displayed a reversed effect: longer RTs for graspable portion-to-hand compatible (414 ms) than for incompatible mapping (390 ms), *t*(19) = 2.835, *p* = 0.011, *d*_z_ = 0.63 (15 out of 20 participants showed their effect in the same direction as the group effect) (Bonferroni-corrected alpha level = 0.025). Thus, as for Experiment 1, the compatibility effect was significantly driven by the location of the spout and not by the task-relevant graspable side. The absolute sizes of the compatibility effects in the two groups did not differ significantly *t*(38) = 0.907, *p* = 0.370, *d*_z_ = 0.29 (see Table 2).

### Post-experiment interview

Results from Experiment 1 and Experiment 2 suggested that the observed compatibility effects were driven by the location of the visually salient spout irrespective of which graspable or goal-directed side of the stimuli was relevant for the task. Since at the end of the experiments participants were asked to report their response strategy, we could evaluate if because of such an attentional bias they were induced to choose a different mapping strategy, or kept the one prescribed in the instructions.

#### Experiment 1

In the compatible mapping blocks, all the participants from the goal-directed and graspable instruction groups reported they applied the S–R mapping of instructions: they attended to the spout and to the graspable side and pressed the button on the same side, respectively. In the goal-directed instruction group—incompatible mapping block (i.e., responses mapped as spatially incompatible to the spout side), 16 participants preferred to attend to the non-salient but compatible graspable portion of the creamers. Two participants, instead, relied on the spout. In the graspable instruction group—incompatible mapping block (i.e., responses mapped as spatially incompatible to the graspable side), all 18 participants reported they remapped after a few trials the incompatible relations for the graspable side to compatible relations for the goal-directed side (e.g., instead of selecting the right button press as the one opposite to the graspable left side, they selected the right button press as the one on the same side of the spout).

#### Experiment 2

As for Experiment 1, in the compatible mapping blocks all the participants from both the groups applied the S–R mapping presented in the instructions.

In the goal-directed instruction group—incompatible mapping block—6 participants relied on the compatible graspable portion of the creamers, whereas 12 participants preferred to attend to the spout, and 2 were unable to report their strategy. In the graspable instruction group—incompatible mapping block—1 participant could not report the strategy, whereas 19 participants reported they remapped after a few trials the incompatible relations for the graspable side to compatible relations for the goal-directed side (e.g., instead of selecting the button press and the grasping action of the right hand as the response opposite to the graspable left side, they selected them as the response on the same side of the spout).

Thus, interviews from both the experiments suggested that the great majority of participants from the graspable instruction groups who were assigned to an incompatible mapping (i.e., 37 over total 38) chose a more economical, compatible mapping strategy based on the location of the salient portion of the stimuli: a behavior consistent with the attention bias produced by this portion of the stimuli, as well as with the absence of affordance activations.

### Combined and distribution analyses

We performed an additional analysis in which we combined the RT data from Experiment 1 and Experiment 2. An ANOVA with experiment and instructions as between-subjects variables and compatibility as within-subjects variable only showed a significant main effect of experiment: overall RTs in Experiment 2 (390 ms) were slower than in Experiment 1 (357 ms), *F*(1, 72) = 14.538, *p* < 0.001, *η*^2^_p_ = 0.17, whereas the interaction between experiment and instructions was not significant, *F*(1, 72) = 3.028, *p* = 0.086, *η*^2^_p_ = 0.04, as well as the interactions between experiment and compatibility, and between experiment, instructions, and compatibility, *F*_s_(1, 72) < 1.9. Not surprisingly, the main effect of experiment reflects the fact that to program a reach-and-grasp action beyond the button press was more time consuming than to program the only button press.

To further investigate the compatibility effects observed in the two experiments, we conducted time-course analyses (Ratcliff 1979). In Pellicano et al. (2017b), time-course investigations (i.e., bin-analyses of RT data) with button press responses displayed nearly flat effect functions, that is, the correspondence effects were already significant at shortest RTs (bin 1) and at all other bins. Indeed, the spatial coding of the task-irrelevant and visually salient side of their stimuli occurred early in time, but then it was also able to exert its influence on those trials for which the participant was taking longer to respond. These results demonstrated that no grasping affordance was active that had a time course different from the salience-driven response activation process (see also Pellicano et al. 2018b). We wanted to investigate if the same was true when the location of the graspable side was relevant for the task, or if affordances could activate and reduced the size of the salience-driven compatibility effect at some point of the RT distribution. In particular, delayed activations of affordances would be better elicited when, in Experiment 2, a grasping action was added to button presses relative to Experiment 1.

Separate bin analyses were run for Experiments 1 and 2. For each participant and compatibility condition, RT distributions were ranked ordered, divided into quintiles (bins) and the mean RT for each quintile was calculated. For each experiment, one ANOVA was run with instructions as the between-participants factor, and bin (from bin1 to bin 5) and compatibility as the within-participant factors. Considering the way that the data were grouped, the bin main effect necessarily turned out to be significant and will not be reported and discussed.

**Fig. 5:**
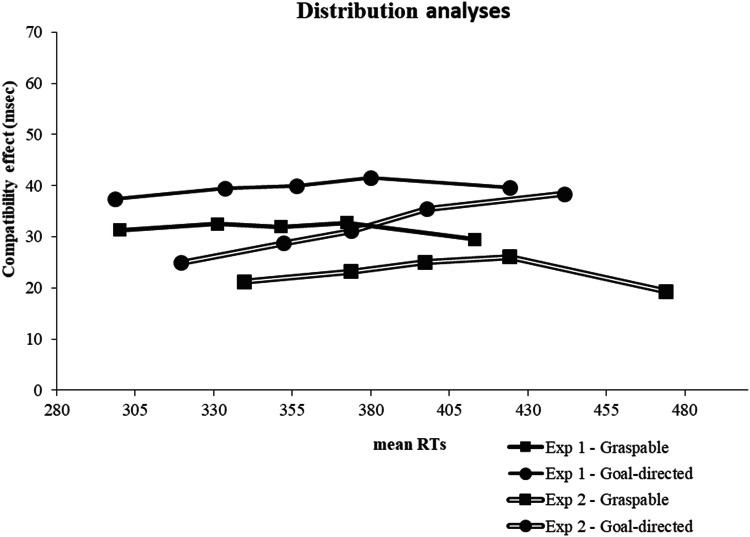
Distribution analyses of RTs in Experiment 1 and Experiment 2. The size of the compatibility effect (in milliseconds) with goal-directed and graspable instructions was plotted as a function of the mean RTs for each quintile. The compatibility effect was displayed as a function of correspondence and non-correspondence between the location of the visually salient portion of the creamers and the location of the response

In Experiment 1, beyond the already discussed main effects and interactions, there was no significant interaction between instructions and bin, *F*(4, 136) = 1.700, *p* = 0.154, *η*^2^_p_ = 0.05. compatibility and bin, *F*(4, 136) < 1, *η*^2^_p_ = 0.01, and between instructions, compatibility and bin, *F*(4, 136) < 1, *η*^2^_p_ = 0.02. In Experiment 2, there was no significant interaction between instructions and bin, *F*(4, 152) = 1.649, *p* = 0.165, *η*^2^_p_ = 0.04. The interaction between compatibility and bin was significant, *F*(4, 152) = 2.537, *p* = 0.042, *η*^2^_p_ = 0.06: the size of the compatibility effect across the five RT bins was: 2, 3, 3, 5, and 10 ms, respectively, and according to Helmert contrasts it did not change from bin 1 to bin 3, *F*(1, 39) < 1.8, *p* < 0.05, tended to increase from bin 3 to bin 4, *F*(1, 39) = 4.064, *p* = 0.051, and increased significantly from bin 4 to bin 5, *F*(1, 39) = 5.115, *p* = 0.029. More informative was the interaction between instructions, compatibility and bin which went close to significance, *F*(4, 152) = 2.360, *p* = 0.056, *η*^2^_p_ = 0.06, and suggested that the above described increasing effect function was mainly due to the goal-directed instruction group. Indeed, this group showed an increasing trend across increasing RT bins, whereas the graspable instruction group displayed a flat effect function of the effect (Fig. 5).

In both the experiments, the time-course analysis excluded that grasping affordances were activated thanks to overt processing of the graspable sides, and with an activation onset different from that of responses activated by the goal-directed side of objects. Indeed, either with button press responses or button press plus reach-and-grasp responses, in both the graspable and goal-directed instruction groups, the compatibility effect was driven by the location of the goal-directed side and its size was not modulated by the speed of reaction times. Not significantly increasing effect functions were produced across the RTs distributions. These results add support to the findings of Pellicano et al. (2017a, b; 2018a; 2018b) on the spatial coding of visual objects based on the side which results to be more salient. More in general, this result is also consistent with studies showing that when variations in the standard Simon task are introduced, a constant (or increasing) correspondence effect is usually found, which is thought to reflect a time-consuming coding of object-inherent spatial codes (see Pellicano et al. 2009).

## Discussion

Since evidence have been provided that spatially distinctive, salient portions of objects can bias motor performance whether or not they consist of the handles, it became crucial to set up experiments able to disentangle the effects of pure salience from those of possible affordance activations. One approach consisted in selecting common use objects with structural features allowing for parallel investigation of the two processes (Pellicano et al. 2017b; 2018b). In Pellicano et al. (2017b), object stimuli had one graspable portion that was not salient to the observer, and conversely a goal-directed, non-graspable portion that was visually salient. In their set of experiments, the location of graspable/goal-directed portions was task-irrelevant, and results clearly showed spatial correspondence effects driven by the location of the salient portion of the objects, instead of the graspable one, thus supporting a location coding account for object-based correspondence effects. Furthermore, Pellicano et al. (2017b) refined the original location coding account claiming that the spatial coding of object tools depends on a higher-level process that implies evaluation of semantic and action features of objects (e.g., depiction of a rightward pouring action), instead of lower-level processing of mere structural asymmetries in objects’ body (see Pellicano et al. 2017b, Experiment 4).

The present study was meant to follow up the original investigation of Pellicano et al. (2017b). In two experiments, we set the location of the graspable and of the goal-directed portions of their creamer stimuli relevant for the task; furthermore, we implemented button press responses (Experiment 1) and button press plus reach-and-grasp responses (Experiment 2). According to the location coding account, instructions to process either the salient or the graspable portion of the stimuli would not modify the amount of their visual distinctiveness and would both produce results similar to the original study: a compatibility effect driven by the spatial location of the salient spout of the creamers. This would happen regardless of the use of simple button presses or the addition of grasping actions. However, if overt attention to the graspable portion alone, or in association with grasping responses was critical to instantiate grasping affordances, participants from the graspable instruction group would show a compatibility effect that depended on the relation between the graspable side of the creamers and the responding hand.

Results of Experiment 1 showed no evidence of affordance activations, but a spatial compatibility effect produced by the location of the spout in both the goal-directed and the graspable instruction groups. Also the time-course analysis displayed a flat effect function across the whole RTs distributions, that is, no grasping actions were competing with the salience-driven responses at any of the RTs bin. Results of Experiment 2 did not differ from those of Experiment 1: performance was still affected by the location of the goal-directed portion of the stimuli in both the instruction groups. The time-course analysis displayed effect functions that were nearly flat across the RTs distributions. Results support previous evidence in Pellicano et al. (2018a) and in Roest et al. (2016) that to employ grasp responses which basically reproduced the grasping action proper for the object stimuli did not foster the activation of affordances compared to the use of simple button presses.

It is worth noting that, as assessed in Pellicano et al. (2017b), chances to activate grasping actions were not reduced in our creamer stimuli, as their graspable side was correctly identifiable even if visual salience was systematically removed from it. Furthermore, in the present study, before each experiment, participants could grasp correctly a real creamer (similar to those displayed as pictures) and performed a proper functional action with it. Nevertheless, no evidence of grasping actions was observed. Rather, a location coding process took place, in both the instruction groups of the two experiments, as driven by the visual asymmetry of the stimuli. Post-experiment interviews suggested that because of this clear asymmetry, the graspable instruction groups adopted by a large majority a more economical S–R mapping strategy: they remapped incompatible relations for the graspable side to compatible relations for the goal-directed side (see also Xiong et al. 2019). Ultimately, the compatibility effect in the graspable instruction groups of Experiment 1 and Experiment 2 reversed and did not significantly differ in size compared to the compatibility effect in the goal-directed instruction groups.

Overall, the results fully supported the prominent role played by perceptual salience and attention mechanisms in object processing. For future investigations on affordances through stimulus–response compatibility paradigms, perceptual salience remains the crucial factor to be controlled to achieve convincing evidence for “pure” limb-specific motor representations rather that abstract response codes. Furthermore, if visual salience of the functional, goal-directed portion of a tool depends on the higher-level coding of its proper action direction (Pellicano et al. 2017b), or similarly, on the representation of a mechanical action (Osiurak et al. 2017), it would be interesting to investigate if this is true not only for object pairs, but also for tools perceived as alone. Indeed, Pellicano et al. (2017b) obtained correspondence effects of similar size and similar time course with objects alone and paired objects, thus claiming some evidence for action coding also for objects alone. In addition, Osiurak et al. (2017) proposed that the mental simulation of a mechanical action between a tool and an object (or maybe also of a tool alone) can guide the perception of the corresponding (hand-centered) affordance: thus to some extent, the mental representation of mechanical actions between objects would precede the activation of affordances between the hand/body and the objects. As reported above, the time-course investigation of correspondence effects performed in this study with task-relevant location of graspable portions, as well as in the previous studies with task-irrelevant location (Pellicano et al. 2017b; 2018a; 2018b), did not show evidence of a later affordance activation “prepared” by the tool-based action coding, but only the direct behavioral effects of the latter. Nevertheless, this point deserves further investigation as a way for possible evidence for pure affordance activations.
